# Circular RNA vaccine, a novel mRNA vaccine design strategy for SARS‐CoV‐2 and variants

**DOI:** 10.1002/mco2.153

**Published:** 2022-07-10

**Authors:** Peng Su, Lei Zhang, Fangfang Zhou, Long Zhang

**Affiliations:** ^1^ Department of Orthopaedic Surgery The First Affiliated Hospital of Wenzhou Medical University Wenzhou PR China; ^2^ Institutes of Biology and Medical Science Soochow University Suzhou China; ^3^ MOE Laboratory of Biosystems Homeostasis and Protection and Innovation Center for Cell Signaling Network Life Sciences Institute Zhejiang University Hangzhou China

1

Qu et al.,^1^ in a paper published in *Cell*, proposed a novel vaccine design strategy that utilizes circular RNA to express the trimeric receptor binding domain (RBD) of the SARS‐CoV‐2 spike protein to elicit antibody responses and cellular immunity in mice and rhesus macaques.^1^ mRNA vaccines can elicit a dose‐dependent antibody response with high virus‐entry inhibition titers and strong Th1‐biased CD4+ T‐cell responses and IFN‐γ+ CD8+ T cell responses.^2^, ^3^ However, mRNA vaccines still have issues, such as insufficient stability at long‐term nonrefrigerator temperatures and production and transportation demands.^4^, ^5^ This study shows the advantages and therapeutic potential of circRNA vaccines against SARS‐CoV‐2 and its emerging variants (Figure 1).

**FIGURE 1:**
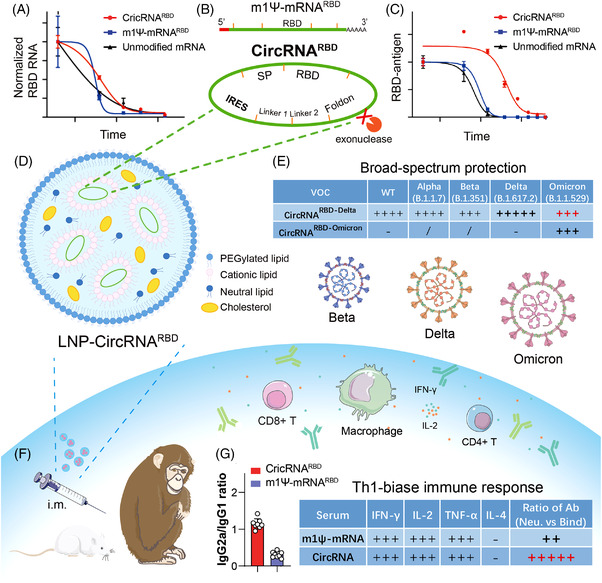
The design strategy and major advantages of circRNA vaccine against SARS‐CoV‐2 and variants. (A and C) Compared with m1Ψ‐mRNARBD, circRNARBD has longer half‐life and more durable antigen expression. (B) IRES element is introduced to initiate the translation of SARS‐CoV‐2 RBD antigen. (D, F, and G) LNP‐encapsulated circRNA vaccines injected intramuscularly into mice and rhesus macaques induce higher proportion of neutralizing antibodies and Th1‐biase immune response than m1Ψ‐mRNA, which is more favorable to SARS‐CoV‐2 clearance. (E) CircRNARBD‐Delta vaccine provides broad‐spectrum protection against both the Delta and Omicron variants. (A), (C), and (E) were reproduced from Ref. 1.

The COVID‐19 pandemic caused by SARS‐CoV‐2 has posed serious threats to public health and led to significant socioeconomic repercussions. In response, mRNA vaccines were rapidly developed and clinical trials were advanced. Several studies have evaluated the immunogenicity of lipid nanoparticle‐encapsulated mRNA (mRNA‐LNP) encoding full‐length SARS‐CoV‐2 spike protein or spike RBD domain, demonstrating that mRNA vaccines, such as mRNA‐1273 and BNT162b1, could elicit robust neutralizing antibodies as well as CD4+ and CD8+ T‐cell responses against SARS‐CoV‐2, to protect the upper and lower respiratory tract from pneumonia.^2^, ^3^ Owing to the high instability of linear mRNA, strict sterility and RNase‐free environment must be ensured during the production process.^5^ A 5` cap, 3` polyA tail, and nucleotide modifications (e.g., 1‐methylpseudouridine, 1mΨ) are necessary to prevent exonuclease digestion, complicating the industrial production of mRNA vaccines.^4^ Moreover, LNP encapsulation employed to improve expression efficiency involves ultracold storage and cold‐chain transportation, which limits its availability in lower resource countries or regions.^5^ Therefore, a better mRNA vaccine design strategy is needed.

Qu et al. proposed the use of antigen‐expressing circular RNA as an LNP‐encapsulated RNA vaccine. Using group I intron self‐splicing or T4 RNA ligase, the authors circularized linear RNA and optimized the in vitro transcription reaction. The internal ribosome entry site element was placed before the RBD‐coding sequence to initiate translation. A secretory signal peptide from human tissue plasminogen activator and trimerization motif (Foldon) of bacteriophage T4 fibritin protein were fused, respectively, to the N‐ and C‐termini of the RBD to ensure the secretion of RBD and improve its immunogenicity. Subsequently, two doses of LNP‐encapsulated circRNAs were intramuscularly injected into the mice at 2‐week intervals. The RBD‐specific IgG endpoint geometric mean titers could reach 106 in the serum. These sera effectively neutralized the SARS‐CoV‐2 pseudovirus and authentic virus in the neutralization assays. These results preliminarily indicated that circRNA vaccines could induce a high level of neutralizing antibody response in vivo.

Further experiments demonstrated two major advantages of circRNA vaccines over linear‐mRNA vaccines. First, the higher the stability, the longer the antigen is expressed. Qu et al. found that circRNA fractions are resistant to exonuclease RNase R. The LNP‐encapsulated circRNA^RBD^, 1mΨ‐mRNA^RBD^, and unmodified mRNA^RBD^ were stored at different temperatures for 1–28 days before antigen detection. The results showed that the antigen expression level in LNP‐circRNA^RBD^ was higher than that in the other two groups. Moreover, LNP‐circRNA^RBD^ induced more beneficial antibody distribution than the other two. ELISA showed that the ratios of IgG2a/IgG1, IgG2c/IgG1, and (IgG2a+IgG2c)/IgG1 from circRNA^RBD^ were consistently higher than those from the 1mΨ‐mRNA^RBD^ vaccines, indicating a higher proportion of Th1‐biased responses, which benefited the clearance of SARS‐CoV‐2. Additionally, LNP‐circRNA^RBD^ induced consistently higher ratios of neutralizing/binding antibodies compared with that by 1mΨ‐mRNA^RBD^ vaccines. Thus, LNP‐circRNA^RBD^ may be better at circumventing the antibody‐dependent enhancement of infection by virus‐specific antibodies.

Given the emerging variants of concern (VOCs) of SARS‐CoV‐2, Qu et al. evaluated the protective efficacies of circRNA^RBD‐Delta^ and circRNA^RBD‐Omicron^ vaccines. The circRNA^RBD‐Delta^ vaccine elicited a higher proportion of neutralizing antibodies than the 1mΨ‐mRNA^RBD‐Delta^ vaccine at 2 and 7 weeks after a booster dose. The third dose of circRNA^RBD‐Delta^ vaccine effectively boosted neutralizing antibodies against both Delta and Omicron variants. The circRNA^RBD‐Omicron^ vaccine induces only Omicron‐specific antibodies. It can be speculated that the circRNA^RBD‐Delta^ vaccine can provide broad‐spectrum protection against current VOCs.

To further evaluate the immunogenicity and protective effects of the circRNA vaccines, rhesus macaques were intramuscularly injected with different doses of circRNA^RBD^ vaccines. High levels of specific IgG were detected in the serum collected 2 weeks after the boost. At 5 weeks after the boost, PBMCs were collected and stimulated with RBD peptide pools to measure the RBD‐specific cell immune responses by ELISpot. There was significant IFN‐γ/IL‐2 production and IL‐4 was undetectable, suggesting that circRNA^RBD^ induced a Th1‐biased response in rhesus macaques. The authors employed SARS‐CoV‐2 authentic virus for challenge experiments and euthanized rhesus macaques 7 days postinfection. RT‐qPCR results showed a nearly 1000‐fold reduction in viral load with protection from circRNA^RBD^ vaccines. Further histopathological examination indicated that the circRNA^RBD^ vaccine effectively protected the lungs of rhesus macaques from the severe symptoms of COVID‐19, such as pulmonary septal thickening and massive inflammatory cell infiltration. These results confirmed the excellent protective ability of circRNA vaccines.

In the face of the long‐troubling COVID‐19 pandemic, mRNA vaccines have been accelerated and applied in clinical treatment, although they have not yet passed the stage of comprehensive and thorough clinical trials. 1‐Methylpseudouridine modification and LNP encapsulation effectively optimized the mRNA stability, immunogenicity, and delivery in vivo. However, the limitations of storage and cold chain transportation due to LNP encapsulation remain to be resolved.

Qu et al. designed circRNA vaccines to try to overcome the above issues. This study provides evidence that circRNA vaccines induce neutralizing antibodies comparable with those induced by 1mΨ‐modified linear RNA vaccines. Due to the inherently high stability and resistance against exonuclease provided, owing to the covalently closed ring structure, circRNA vaccines can synthesize and release antigens in vivo, while inducing Th1‐biased cellular immunity responses. Although in this research, circRNA vaccines were not compared with two approved mRNA vaccines (mRNA1273 or BNT162b2) that have been used in large‐scale vaccination drives, they still give vaccine developers new ideas for improving mRNA vaccines, which is significant for the prevention and control of COVID‐19 and other viral pandemics that may emerge in the future.

## CONFLICT OF INTERESTS

The authors declare that they have no conflict of interest.

## ETHICS STATEMENT

Not applicable.

## AUTHOR CONTRIBUTION

Peng Su and Lei Zhang conducted the literature review and drafted the manuscript, Fangfang Zhou and Long Zhang revised the manuscript.

## Data Availability

Not applicable.
